# *Cassiopea xamachana* polyp feeding under husbandry conditions

**DOI:** 10.17912/micropub.biology.001561

**Published:** 2025-04-15

**Authors:** Victoria Sharp, Kendra Pfeil, Kaitlin Kitch, Mónica Medina

**Affiliations:** 1 Pennsylvania State University, State College, Pennsylvania, United States

## Abstract

Research on the upside-down jellyfish *Cassiopea xamachana* has increased in the past few decades, hence the need for more efficient husbandry protocols. We tested the effect of weekly feeding frequencies, light cycles, and nutrient supplements on symbiotic and aposymbiotic polyp asexual reproduction and mortality. *C. xamachana* polyps have better survivorship and reproduction when kept in a day/night cycle and given additional food beyond *Artemia* nauplii.

**Figure 1. Asexual reproduction strategies of Cassiopea xamachana .**
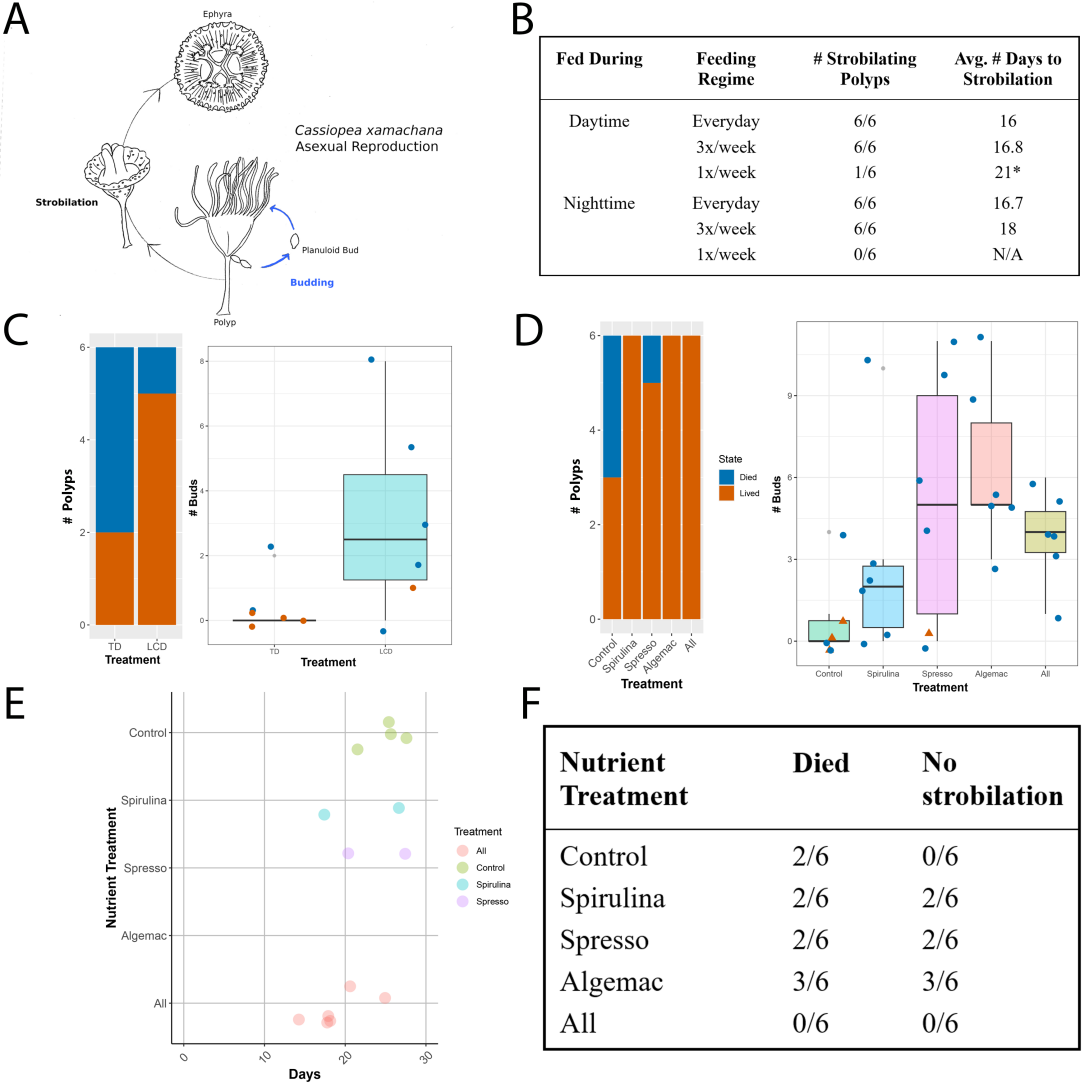
Figure 1. A) Asexual reproduction strategies of *Cassiopea xamachana* . Buds detach from budding chains and settle into new polyps, as shown by the blue arrows. Strobilation shown by black arrows. B) Strobilating polyps and average number of days to strobilation for symbiotic polyps under different light and feeding regimes. C) Number of buds produced by polyps kept in different light conditions. TD= Total Darkness, LCD= 12 hour Light/Dark Cycle. Left shows number of polyps that lived (orange) or died (blue). Right shows box plot of number of buds produced by treatment with each dot representing an individual polyp, colored by lived (orange) or died (blue). Small gray dots indicate outlier data points. D) Survivorship and number of buds produced per nutrient treatment. Left shows number of polyps that lived (orange) or died (blue). Small gray dots indicate outlier data points. Right shows box plot of number of buds produced by treatment with each dot representing an individual polyp, colored by lived (orange) or died (blue). All nutrient treatments had a higher survivorship and number of buds produced compared to control. E) Number of days to strobilation for polyps fed the different nutrient treatments. The polyps had been fed nutrient treatments for a minimum of 2 weeks prior to being given symbionts. F) Record of polyps that died or did not strobilate after providing symbionts.

## Description

In the jellyfish *Cassiopea xamachana* juvenile polyps have garnered interest among developmental biologists and naturalists due to their asexual reproduction and as the key developmental stage for the transition from aposymbiotic to symbiotic lifestyle (Ohdera et al. 2018). *C. xamachana* polyps cannot undergo strobilation and metamorphose into adult medusa without the presence of a photosynthetic endosymbiont (Ohdera et al. 2018; Newkirk et al. 2018; Newkirk et al. 2020; Sharp et al. 2024) from the dinoflagellate family Symbiodiniaceae (LaJeunesse et al 2018). ( Figure 1A ). Husbandry practices on polyps are not well-described in the literature. Commonly, researchers have kept aposymbiotic polyps in dark conditions as a precaution to prevent potential photosymbiont proliferation in the case of seawater contamination (Fitt and Trench, 1983; Newkirk et al. 2018; Sharp et al. 2024). Adult *C. xamachana* ’s circadian rhythm is disrupted in complete darkness displaying abnormal pulsing and feeding behavior (Nath et al. 2017), suggesting polyps also need a day/night cycle to maintain homeostasis. As demonstrated in mice, these slight changes in daily care can significantly impact animal wellness (Gerdin et al. 2012). In invertebrates like *C. xamachana* , where stress is not as easily measured, animal wellness can be captured by mortality and reproduction counts.

In this study we explore different husbandry conditions of *C. xamachana* polyps to evaluate rates of animal production. Time of day and frequency of feeding was tested against speed of asexual reproduction in polyps. We wanted to explore if polyps can feed during the night portion of their diurnal light cycle and the ideal weekly feeding frequency of jellyfish to maintain a population. Aposymbiotic polyps were kept in a day/night cycle or perpetual darkness to explore if a circadian rhythm affects polyp health. Once a consistent feeding strategy and light protocol was established, we examined diet treatment. Many aquaculture facilities feed *Artemia* to scyphozoans (AZA Aquatic Invertebrate TAG 2021), and the *Artemia* are fed blends of phytoplankton or other supplements to provide additional nutrients for the jellyfish. We evaluated nutrient supplements of an *Artemia* diet based on the diet provided to moon jellyfish at the Newport Aquarium (Oregon, USA) as follows: (1) ‘Control’, (2) *‘Spirulina’* , (3) ‘Spresso’, (4) ‘Algemac’, (5) ‘All’ (see Reagents). Feeding these supplements to *Artemia* ensures bioaccumulation of nutrients in the jellyfish while minimizing waste accumulation in the jellyfish water (Watanabe et al. 1982).

When polyps were fed during the day or night at different weekly frequencies (everyday, three times a week, or once a week), only polyps in the once a week group died regardless of day or nighttime feeding ( Figure 1B ). The once a week feeding regime failed to result in any polyps strobilating. Over half of the polyps kept in perpetual darkness died, while only one polyp kept in a day/night cycle died ( Figure 1C ).

For nutrient treatments, blends (see Reagents) were first provided to *A. fransiscana* prior to feeding to *C. xamachana* . The average number of buds produced per polyp was highest for the ‘Algemac’ treatment (6.333 buds) compared to the control of 0.833 buds, and the ‘Algemac’ treatment produced 38 total buds compared to 5 total for the control ( Figure 1D ). The nutrient treatment of *‘Spirulina’* produced the fewest number of buds for all treatments (17 total, 2.833 per polyp)( Figure 1D ). ‘Spresso’ had the widest range of bud production and was also the only treatment to not provide extra protein (protein content can be found on website for ‘Spresso’ product listed in Reagents)( Figure 1D ). Considering the energy likely required for asexual reproduction, additional protein may facilitate bud production. No polyps died during this part of the experiment however, several polyps died after symbionts were introduced to the polyps ( Figure 1E ). Several polyps also did not strobilate during the 30-day observation period ( Figure 1F ). No polyps strobilated for the ‘Algemac’ treatment, which also had the highest polyp mortality of 50% ( Figure 1E ). Every polyp strobilated and survived with the ‘All’ treatment ( Figure 1F ).

Our collection of experiments gives a precursor look into ideal husbandry conditions for *C. xamachana* polyps to maximize asexual reproduction in healthy laboratory populations. We have shown that a day/night cycle lowers polyp mortality compared to a continuous dark cycle, and that frequency of feeding affects the ability of polyps to successfully strobilate. Feeding once a week with *A. fransiscana* nauplii is not sufficient to induce strobilation compared to feeding three times a week or every day. Feeding every day appears to lower the amount of time it takes to induce strobilation, which increases animal reproductive success and the production of animals for laboratory work. Even when fed three times a week, aposymbiotic polyps experienced higher mortality when kept in darkness as compared to experiencing a day/night cycle ( Figure 1C ). Taken together, this suggests that feeding three times a week and maintaining a day/night cycle is the minimum optimal setup to ensure a healthy polyp colony. Time of feeding did not significantly impact polyp mortality or time to strobilation, and polyps appear able to feed even during a nighttime period.

Nutrient supplements have a positive effect on aposymbiotic polyp budding and symbiotic polyp strobilation. It appears the “All” treatment had a consistent benefit to polyp reproduction, as it produced a large number of buds and induced strobilation faster than the control ( Figure 1D, 1E, 1F). The “All” nutrient combination had zero polyp mortality upon introduction of the symbionts, while the other treatments exhibited polyp death ( Figure 1F ). There were multiple organic compounds in the nutrient supplements that are not found in *Artemia* nauplii (see Reagents for nutrient blends used), and it appears the addition of caratenoids, vitamins, docosahexaenoic acid (DHA), eicosapentaenoic acid (EPA), butylated hydroxyanisole (BHA), and butylated hydroxytoluene (BHT) may be crucial compounds for increased *C. xamachana* asexual reproduction. We saw an increase in survivorship and reproductive success in *C. xamachana* polyps, showing that the addition of nutrients to *Artemia* cultures aid researchers achieve abundant jelly populations.

To keep research comparable, it is essential to standardize *C. xamachana* husbandry. These experiments aid the scyphozoan community in ensuring healthy animals and provides suggestions for increasing laboratory animal populations. Altogether, the work presented here shows the need for optimizing *C. xamachana* husbandry efforts for consistency in animal welfare and experimental results.

## Methods

Polyps were kept in six-well CellStar culture plates (Thomas Scientific) with one polyp per well in approximately 7 mL of 35 ppt artificial seawater (Instant Ocean, Spectrum Brands, Inc.). Polyps were fed 2-10 one-day old *A. fransiscana* nauplii, and water changes occurred three times a week.

For studying the effect of feeding regime, six feeding schedules were created; (1) feeding once a week in the day cycle, (2) feeding three times a week in the day cycle, (3) feeding every day in the day cycle, (4) feeding once a week in the night cycle, (5) feeding three times a week in the night cycle, (6) feeding every day in the night cycle. Polyps were acclimated to feeding schedules for 2 weeks before given symbionts and kept in a 12:12 day/night cycle of 27.3°C/26.5°C at a light intensity of 150 μmol/m2/s. Approximately 250,000 cells/mL of the dinoflagellate symbiont *Symbiodinium microadriaticum* were given to each polyp on the same day, followed immediately by feeding with *A. fransiscana* to encourage ingestion of the photosymbionts. Feeding of each polyp occurred approximately 1 hour after the respective day/night cycle began. The number of days from introduction of symbiont to induction of strobilation was recorded, along with ephyra and polyp death.

Aposymbiotic polyps were observed kept in perpetual darkness or on a day/night cycle. Polyps in the ‘dark’ treatment were kept in an incubator at 26.5°C in the dark continuously, except for the approximately 5 minutes it took for water changes and feeding. Polyps in the ‘light’ treatment were kept as described above. Polyps were fed three times a week. Polyps were allowed to acclimate to their conditions for 2 weeks before data was recorded. The number of buds produced and polyp death was recorded for 31 days.

Diet was explored with aposymbiotic and symbiotic polyps. The blends described in Reagents were added to clean containers with a bubbler and one-day old *A. fransiscana* nauplii. The nauplii were left for 4 hours, strained out of the media, and transferred to the polyps for feeding. The polyps were kept in a day/night cycle as described previously. Polyps were acclimated to the light cycle for 2 weeks prior to starting nutrient treatments. Buds produced per polyp were counted for 31 days. At the end of the 31 days, all polyps were then given approximately 250,000 cells/mL of *S. microadriaticum* CassKB8. The number of days for successful detachment of an ephyra was then recorded as number of days until strobilation.

**Statistical analysis**

All statistical analysis was run in R (R Core Team 2018). For polyps fed during the day or night at different weekly frequencies, a One-Way ANOVA was run on the number of days to strobilation for polyps fed in day or night (p=0.1744), polyps fed at different weekly frequencies (p=0.4596), or the interaction of time of day and weekly frequencies (p=0.1972 and 0.3229 respectively). None of these tests achieved a significance cutoff of p < 0.05. treatments. A two-sample t-test on the number of buds produced by polyps kept in perpetual darkness or with a day/night cycle (p=0.03193). This achieved significance (p < 0.05) as dark polyps produced fewer buds by far, and 4 of 6 polyps died in the exclusively dark treatment. A One-Way ANOVA on the number of buds produced by each nutrient treatment (p=0.05666) did not achieve the significance cutoff of p < 0.05. A One-Way ANOVA of the number of days to strobilation between the different nutrient treatments did not achieve a significance cutoff of p < 0.05 (p=0.4929).

## Reagents

*C. xamachana* polyps for each experiment came from the T2D clonal line of the Medina Lab (Pennsylvania State University, USA).

The dinoflagellate *Symbiodinium microadriaticum* strain used for symbiosis induction was CassKB8.

Six-well CellStar culture plates (Thomas Scientific) were used to hold polyps.

Instant Ocean, Spectrum Brands, Inc. were used to make artificial seawater.

Nutrient supplements were used with source company listed:

**Table:** 

**Name**	**Ingredient**	**Quantity**	**Concentration**	**Source**
**Control**	*Artemia fransiscana*	3-10 nauplii	NA	Brine Shrimp Direct
**Spirulina**	Spirulina Powder ( *Arthrispira platensis* )	~0.02%	0.02%	Brine Shrimp Direct
**Spresso**	S.presso	½ drop / 50 mL SW	0.0006%	INVE Aquaculture
**Algemac**	Liquid Red Algamac-DHA30	1.5 drops/50 mL	0.002%	Aquafauna Bio-Marine
	Spresso	0.5 drops/50 mL	0.0006%	INVE Aquaculture
	Easy DHA Selco	0.5 drops/50 mL	0.0006%	INVE Aquaculture
**All**	Spirulina Powder ( *Arthrispira platensis* )	5 mL of each treatment	0.02%	Brine Shrimp Direct
	*Pavlova sp* . Cryo-Preserved Algae Paste	1 drop	0.006%	Brine Shrimp Direct
	Liquid Red Algamac-DHA30	5 mL of each treatment	0.002%	Aquafauna Bio-Marine
	S.presso	5 mL of each treatment	0.0006%	INVE Aquaculture
	Easy DHA Selco	5 mL of each treatment	0.0006%	INVE Aquaculture
